# Physical Activity Alleviates Cognitive Dysfunction of Alzheimer’s Disease through Regulating the mTOR Signaling Pathway

**DOI:** 10.3390/ijms20071591

**Published:** 2019-03-29

**Authors:** Xianjuan Kou, Dandan Chen, Ning Chen

**Affiliations:** 1 Tianjiu Research and Development Center for Exercise Nutrition and Foods, Hubei Key Laboratory of Exercise Training and Monitoring, College of Health Science, Wuhan Sports University, Wuhan 430079, China; kouxianjuan@126.com; 2 Graduate School, Wuhan Sports University, Wuhan 430079, China; chendandan950310@163.com

**Keywords:** Alzheimer’s disease, autophagy, mTOR signal pathway, physical activity, microRNA

## Abstract

Alzheimer’s disease (AD) is one of the most common aging-related progressive neurodegenerative disorders, and can result in great suffering for a large portion of the aged population. Although the pathogenesis of AD is being elucidated, the exact mechanisms are still unclear, thereby impeding the development of effective drugs, supplements, and other interventional strategies for AD. In recent years, impaired autophagy associated with microRNA (miRNA) dysfunction has been reported to be involved in aging and aging-related neurodegenerative diseases. Therefore, miRNA-mediated regulation for the functional status of autophagy may become one of the potent interventional strategies for AD. Mounting evidence from in vivo AD models has demonstrated that physical activity can exert a neuroprotective role in AD. In addition, autophagy is strictly regulated by the mTOR signaling pathway. In this article, the regulation of the functional status of autophagy through the mTOR signaling pathway during physical activity is systematically discussed for the prevention and treatment of AD. This concept will be beneficial to developing novel and effective targets that can create a direct link between pharmacological intervention and AD in the future.

## 1. Introduction

Alzheimer’s disease (AD) is an insidious, age-dependent progressive neurodegenerative disorder characterized by deficits in cognitive function. The pathological changes of AD are diffuse atrophy of the cerebral cortex, deepening of cortical sulci, and narrowing of cerebral gyri, in which the loss of neurons, the extracellular deposition of amyloid-beta (Aβ) peptide as senile plaques (SPs), and the formation of neurofibrillary tangles (NFTs) are characteristic [1,2]. Up to now, a series of studies on the pathogenesis of AD have been conducted, and several hypotheses including Aβ cascade [3], abnormal tau phosphorylation [4], increased apolipoprotein E (APOE) [5], and neuroinflammation [6] have been widely recognized. However, no hypothesis has been completely elucidated on the complex pathological changes of AD.

Autophagy as an evolutionary-conserved process can maintain normal physiological events or regulate the progression of a series of diseases through sequestering mis-folded/toxic proteins in autophagosomes, thus executing its cytoprotective role [7,8]. Growing evidence demonstrates that autophagic capacity to degrade harmful proteins in cells declines with increasing age [9,10]. Moreover, dysfunctional autophagy has also been linked to several aging-related neurodegenerative diseases including AD [11,12,13,14,15,16,17,18,19]. Previous studies have documented the critical role of autophagy in the pathogenesis of AD, including Aβ production or deposition, Aβ precursor protein (APP) metabolism, and neuronal death [20,21]. Furthermore, insufficient or reduced autophagic activity can lead to the formation of harmful protein aggregates, which results in increased reactive oxygen species (ROS), cell death, and neurodegeneration [22]. As a result, autophagy has a crucial role in the regulation of longevity.

Mammalian target of rapamycin (mTOR) regulates a series of physiological processes. On the one hand, mTOR plays an important role in different cellular processes including cell survival, protein synthesis, mitochondrial biogenesis, proliferation, and cell death [23,24]. On the other hand, the mTOR signaling pathway can execute an important role in memory reconsolidation and maintaining synaptic plasticity for memory formation, due to its regulatory function for protein synthesis in neurons [25]. Moreover, mTOR also can interact with upstream signal components, such as growth factors, insulin, PI3K/Akt, 5′-adenosine monophosphate-activated protein kinase (AMPK), and glycogen synthase kinase 3 (GSK-3) [26,27]. Currently, although the molecular mechanisms responsible for AD remain unclear, more and more studies have confirmed the involvement of dys-regulated mTOR signaling in AD [28,29]. Activated mTOR signaling is a contributor to the progression of AD and is coordinated with both the pathological and clinical manifestations of AD [30]. Furthermore, there is a close relationship between mTOR signaling and the presence of Aβ plaques, NFTs, and cognitive impairment in clinical presentation [31,32,33]. Therefore, the development of mTOR inhibitors may be useful for the prevention and treatment of AD.

It has been reported that regular physical activity can improve brain health and provide cognitive and psychological benefits [34]. Mechanically, regular exercise training is related to the inhibition of oxidative stress and apoptotic signaling, thus effectively executing neuroprotection [35]. Previous studies have demonstrated that treadmill or voluntary wheel running is beneficial for the improvement of behavioral capacity, and can promote the dynamic recycling of mitochondria, thereby improving the health status of mitochondria in brain tissues [36]. Moreover, other studies have demonstrated that regular exercise has a beneficial effect on the structure, metabolism, and function of human and rodent brains [37,38]. Interestingly, our recent study has also documented that the brain aging of d-gal-induced aging rats can be noticeably attenuated by eight-week swimming training, due to the rescuing of impaired autophagy and abnormal mitochondrial dynamics in the presence of miR-34a mediation [39]. Therefore, physical activity is regarded as an effective approach against AD. The aim of this article is to overview the potential of physical activity as a preventive or therapeutic strategy for AD through regulating the mTOR signaling pathway. In this article, we summarize the main features of AD pathogenesis, the regulatory roles of mTOR in AD, and the preventive or therapeutic implications of targeting the mTOR signaling pathway with physical activity or exercise intervention.

## 2. The mTOR Signaling Pathway

### 2.1. The mTOR Signaling Pathway and Autophagy

mTOR can be divided into two different functional complexes: mTOR complex 1 (mTORC1) and mTOR complex 2 (mTORC2). These mTOR complexes are localized in the center of complex signaling pathways that are activated by growth factor signals or intracellular stress. mTOR can undergo self-phosphorylation via its own serine/threonine kinases, and can regulate the synthesis of other proteins by activating p70-S6K phosphorylation [40]. Similarly, as the first downstream substrate of mTOR, 4E-BP1 is a translational repressor that inhibits the translation initiation associated with eukaryotic translation initiation factor 4E (eIF4E). Under normal conditions, 4E-BP1 presents in a de-phosphorylation state in combination with eIF4E to form a complex. Under the stimulation of growth signals, 4E-BP1 can be inactivated due to the phosphorylation of mTOR, and p-4E-BP1 can be detached from eIF4E, thereby losing the inhibition of eIF4E [41]. When mTOR is activated, it can phosphorylate its key downstream molecules such as 4EBP1 and S6K1 to promote protein synthesis [42]. Furthermore, mTOR is also involved in the regulation of autophagy. Previous studies have demonstrated that the hyperactivation of mTOR can reduce autophagy and directly contribute to hyperphosphorylation and aggregation of tau protein [43,44].

Two mTOR complexes have different sensitivity to rapamycin. The mTORC1 is a rapamycin-sensitive complex and the mTORC2 is a rapamycin-independent complex. The mTORC1 can inhibit autophagy under the condition of sufficient nutrients and energy through phosphorylating Unc51-like kinase 1 (ULK1) and autophagy-related gene 13 (Atg13), which is essential for the formation of pre-autophagosomal structures [45]. Usually, mTOR regulates autophagy [46]. The inhibition of mTORC1 induces autophagy while its activation suppresses autophagy. Consistent with a previous study, the treatment with rapamycin in Alzheimer’s transgenic mice (P301S mice) activates autophagy and suppresses tau hyperphosphorylation to prevent the aggregation of tau protein [47]. Therefore, mTOR inhibitors may have a protective role against AD. In addition, prolonged rapamycin treatment can inhibit Akt activity in many types of cells by suppressing mTORC2 assembly [48]. Since Akt positively regulates mTORC1, the phosphorylation of Akt by mTORC2 can stimulate the function of mTORC1, thereby inhibiting autophagy. In addition, mTOR also regulates protein synthesis in neurons at the translational level by phosphorylating several intracellular targets. One finding in invertebrates indicates that mTOR-dependent translational control is critical for synaptic plasticity and learning and memory reconsolidation [25]. The studies using various models have also confirmed mTOR as a critical signaling pathway for synaptic plasticity [49]. Considering its regulatory roles, mTOR could be a promising target for suppressing the neurodegenerative process and rescuing the adult brain from pathological changes.

A series of studies have demonstrated that the activation of autophagy can exert a neuroprotective function; in contrast, deficient autophagy or impaired autophagic flux can result in neurological damage in most neurological disorders [21,50,51]. For example, the deficiency of autophagy-related gene *Beclin1* in cultured neurons and transgenic mice provokes the deposition of Aβ, whereas its overexpression attenuates accumulation of Aβ [18]. Growing evidence has shown that lysosomal system defects are the key pathogenic factors in AD; thus, selectively restoring lysosomal function in mouse AD models can alleviate deficient cognitive capacity and synaptic function [52,53]. It has been reported that autophagic flux is altered in patients with AD, and the administration of autophagy enhancer rapamycin may alleviate cognitive impairment and Aβ neuropathology in APP/PS1 mouse models [54]. Consistent with these opinions, one recent report [55] has demonstrated that autophagic sequestration is stimulated in patients at the early stage of AD, while lysosomal clearance is progressively declining and autophagic flux is gradually hindered due to the lack of the substrate clearance. Previous studies have shown that rapamycin, a selective inhibitor of TORC1, can attenuate Aβ accumulation and inhibit tau phosphorylation in AD mouse models [56]. On the contrary, mTORC2 seems to indirectly suppress autophagy through phosphorylating Akt, thereby resulting in the activation of Akt/mTORC1 signaling [57]. Recent studies have also demonstrated that chronic intervention using rapamycin can retard the progression of AD-like deficits and decrease Aβ level by inducing autophagy in the mouse model with overexpression of human APP [58].

### 2.2. Activated mTOR Signaling Triggers Aβ Generation and Induces the Failure of Aβ Clearance

AD is a progressive neurodegenerative disease caused by the accumulation of toxic proteins that leads to neural damage and cell death [51]. A large number of studies have shown that the activation of mTOR is an enhancer of Aβ generation and deposition [31,59]. Under normal conditions, Aβ is degraded by the autophagic-lysosomal pathway, thus participating in protein quality control and the removal of aberrant forms of protein. mTOR also modulates the metabolism of APP by regulating β- and γ-secretases. Different animal and cell models have also provided evidence that excessive mTOR activity increases the activity of β- and γ-secretases, thus leading to the generation of Aβ plaques and the activation of mTOR related to the malfunction of Aβ elimination from the brain, since mTOR-mediated inhibition of autophagy could lead to the accumulation of Aβ [33,54,60]. The role of mTOR-dependent autophagy dysfunction has been previously reported in a variety of neurological and neuropsychiatric disorders [61,62,63,64,65]. Increasing studies have proven that mTOR activation leads to the failure of Aβ removal from the brain, since the dysfunction of autophagy triggered by mTOR facilitates the process of Aβ generation and weakens its clearance [33,54,60]. In the 3xTg mouse model with AD, autophagy induced by rapamycin has been reported to ameliorate cognitive deficits through inhibiting mTOR signaling [66]. Chronic treatment with rapamycin reduces the progression of AD by inducing autophagy, which, in turn, reduces Aβ level in the mouse model with human APP [58]. Apart from this, the relationship between the immaturity of autophagolysosomes and the accumulation of autophagic vacuoles (AVs) that can contribute to the generation of Aβ has also been confirmed. In this case, the activation of mTOR signaling alters the autophagic process, thus leading to the accumulation of immature forms of AVs [67].

### 2.3. mTOR Activation Induces Hyperphosphorylation of Tau Protein

Tau is a microtubule-binding protein that promotes microtubule assembly and stabilization to form a stable cytoskeletal system. In contrast, the ability of hyperphosphorylated tau protein (pathological tau protein) to bind to microtubules is significantly reduced, thereby losing the ability to promote microtubule assembly and maintain microtubule stability, disrupting the cytoskeletal system, and impairing the normal function of neurons [4]. Wild-type tau in vivo can result in synaptic loss, whereas the deletion of tau can rescue Aβ-induced neurotoxicity at the synapse [68,69,70]. The chronic stress and mTOR-dependent inhibition of autophagy can lead to the accumulation of tau aggregates in P301L-tau-expressing mice and cells, which is validated by molecular, pharmacological, and behavioral analysis [71], suggesting that dys-regulated generation, phosphorylation, and aggregation of tau might be the key events for triggering neuronal degeneration in AD. Currently, little is known about the upstream intracellular effectors accounting for these molecular events in the process of tau deposition, but mTOR has been proposed. The signaling pathway mediated by mTOR kinase regulates protein homeostasis via facilitating protein translation [47]. The abnormal mTOR signaling can be observed in an AD brain [72]. Recent evidence indicates that tau can mediate learning and memory deficits in animal models with AD [68], suggesting that reducing tau level may represent a valid therapeutic approach. mTOR and its downstream p70S6K have been reported to be higher in human AD brains [73].

Growing evidence has shown that mTOR links to aging from lower organisms to mammals. For example, genetically increasing mTOR signaling can upregulate tau level and promote tau phosphorylation, but reducing mTOR signaling with rapamycin can ameliorate tau pathology and rescue motor deficits in a mouse model of tauopathy [74,75]. Consistent with in vivo experiment, in vitro results suggest that mTOR signaling regulates tau phosphorylation [43] and the activation of mTOR enhances tau-induced neurodegeneration in a Drosophila model of tauopathy [76]. Tau phosphorylation is dynamically regulated by mTOR. Numerous scientific data support the key role of mTOR in the tau-related pathological progress, thus implying that the activity of mTOR determines the abnormal hyperphosphorylation of tau and the formation of NFTs [47,56]. mTOR signaling activation increases abnormal phosphorylation of tau, while inhibiting mTOR attenuates abnormal phosphorylation of tau. Consistent with the above reports, a transgenic mouse model subjected to treatment with rapamycin revealed alleviated cognitive impairment and reduced accumulation of Aβ plaques and NFTs due to the induction of autophagy [77,78]. Therefore, mTOR is an effective preventive or therapeutic target for AD by regulating tau phosphorylation and controlling the autophagic signal pathway.

## 3. The Alteration of miRNAs in AD and Aging-Related Diseases

MicroRNAs (miRNAs), small non-coding RNAs with a length of 18–25 nucleotides, usually downregulate the expression of mRNA and protein upon targeting specific mRNAs, and are involved in complex post-transcriptional regulatory networks and the maintenance of healthy cellular functions [79,80,81,82]. Approximately 70% of known miRNAs enriched in the brain are involved in critical roles, including neuronal development and differentiation, synaptic plasticity, and the pathogenesis of neurodegenerative disorders [83]. The expression of some miRNAs is dynamically regulated during brain development, neurogenesis, and neuronal maturation [84]. In recent years, growing evidence has demonstrated that abnormal patterns of miRNAs are linked with most aging or aging-related neurodegenerative diseases [83,85]. In APP/PS1 mice, miR-99b-5p and miR-100-5p are reported to be decreased and increased at early and late disease stages compared with age-matched wild-type mice, respectively [86]. In addition, miR-99b-5p and miR-100-5p are reported to affect neuron survival by targeting mTOR, which is consistent with previous studies in cancer [87,88,89]. The defensive effect of miR-200b or miR-200c on Aβ-induced toxicity in AD models are observed, which is evidenced by the relieving of impaired spatial learning and memory induced by intracerebroventricular injection of oligomeric Aβ after the treatment of miR-200b or miR-200c [90]. Mechanically, the miR-200b/c could suppress the downstream effector of mTOR, S6K1. Chronic cerebral hypoperfusion (CCH) is a high-risk factor for vascular dementia and AD. Similar to a previous study, some miRNAs have also been validated to regulate autophagy-related signal pathways [39]. It is reported that the level of miR-96 is significantly increased in a CCH rat model established by two-vessel occlusion (2VO), and the inhibition of miR-96 can attenuate the cognitive impairment. Furthermore, miR-96 antagomir injection can attenuate the number of LC3 and Beclin1-positive autophagosomes in 2VO rats. In contrast, the overexpressed miR-96 can downregulate mTOR protein levels in 2VO rats and primary culture cells [91]. These findings suggest that miR-96 may play a key role in autophagy under CCH by regulating mTOR signaling. Since pathological changes occurring in AD and Parkinson’s diseases (PD) brains are reflected in cerebrospinal fluid (CSF) composition, CSF represents an optimal biomarker source of neurodegenerative diseases. One study [92] related to CSF miRNAs has reported that 74 miRNAs are downregulated and 74 miRNAs are upregulated in AD patients when compared with controls based on a 1.5-fold change threshold. The study identified a set of genes involved in the regulation of tau and Aβ signal pathways in AD, with mTOR and BACE1 being targeted by the CSF miRNAs. Another study [93] has demonstrated that miR-153, miR-409-3p, miR-10a-5p, and let-7g-3p are significantly overexpressed in CSF exosomes from PD and AD patients. Bioinformatic analysis has demonstrated that mTOR signaling, ubiquitin-mediated proteolysis, dopaminergic synapses, and glutamatergic synapses are the most prominent pathways, with differential exosomal miRNA patterns associated with the development of PD and AD. These results have demonstrated that CSF miRNA molecules are reliable biomarkers with fair robustness in regard to specificity and sensitivity in differentiating PD and AD patients from healthy controls. Among these processes, the mTOR signaling pathway is an important target.

The roles of miRNAs in APP and Aβ production, synaptic remodeling, neuron survival, and glia cell activation have also been identified [94,95]. miRNAs, including miR-130a, miR-20a, miR-29a, miR-106b, miR-128a, miR-125b, and miR-let-7c, have been reported to be downregulated in aged individuals and in different human and animal cell aging models [85,96,97]. In the brain, miR-29 is reported to target BACE1, and the deregulation of miR-29b results in an increase of apoptosis in AD. The overexpression of miR-29 in humans and transgenic mice could decrease endogenous BACE1 levels and increase Aβ production [79]. MiR-107 also targets BACE1, and can induce cell cycle arrest, because cell cycle re-entry is an early event in AD pathogenesis [98]. Of course, there are some brain-specific miRNAs that participate in tau hyperphosphorylation, the physiological regulation of APP expression, and the generation and deposit of Aβ. The expression of extracellular signal-regulated kinase 1 (ERK1) is a direct tau kinase. Some miR-15 family members can target ERK1 to be involved in tau hyperphosphorylation [79]. For example, as a neuron-specific miRNA, the expression of mature miR-124 is reduced in a subset of AD patients [99]. Downregulation of miR-124 can result in the altered splicing of APP and promote the conversion of APP to Aβ. Similarly, the downregulation of miR-17, miR-101, and miR-16a also promotes accumulation of APP [33,37]. Previous studies have documented that the abnormally low expression of miR-16 could potentially lead to the accumulation of APP protein in the embryo of SAMP8 mice and BALb/c mice, suggesting APP as a target of miR-16 [100]. The miR-101 and miR-106 can also target APP, in turn, resulting in an elevated generation and accumulation of Aβ [101]. miR-455-3p is found to be significantly upregulated in serum samples, postmortem brains, mouse models, and cell lines of AD [102]. Recent evidence has shown that circulating miR-455-3p is upregulated in AD postmortem brains when compared with healthy control samples [81], suggesting that miR-455-3p may be a potential biomarker for AD. The miR-206 regulating brain-derived neurotrophic factor (BDNF) is markedly increased in AD model mice [103]. Because changes in gene expression and splicing of APP are associated with the generation and deposition of Aβ, specific neuronal miRNAs can regulate APP splicing. Therefore, the scanning and identification of miRNAs in the future could provide an important new insight and elucidation in the initiation and progression of AD. Nevertheless, the roles of far more miRNAs still remain enigmatic in AD etiology.

## 4. The Role of Physical Activity in AD

Physical activity not only affects skeletal muscle, but also has an important effect on the phenotype of the brain. The brain has high sensitivity to exercise, so that exercise in rodent models is easy to drive the neurogenesis within hippocampal and dentate gyrus (DG) areas, thus leading to enhanced learning and memory capabilities. Defining the optimal preventive strategy according to type, duration, and intensity of physical activity is a key practical question. In this article, the roles of physical activity as a potential preventive intervention against AD are summarized, which will be beneficial to exploring optimal exercise prescriptions for the prevention and treatment of AD, and providing references for developing novel and effective targets for the prevention and treatments of AD in the future.

### 4.1. Physical Activity is Beneficial for the Improvement of Learning and Memory Capacity

Cognitive decline has increasingly been reported in correlation with human aging [104]. This age-related decline of cognitive capacity also occurs in mice [105]. Some animal studies [106,107,108] demonstrate that cognitive impairment can occur in the absence of Aβ deposition and NFTs. However, several researchers have shown that a physically active lifestyle can modify cognitive decline in both humans and mice [109,110]. Moreover, regular physical activity can improve brain health and provide cognitive and psychological benefits. Physical activity has been shown to improve mental health and cognition, including in patients with AD [34]. Regular exercise may also improve different cognitive domains, such as memory and executive function, in older-age individuals with dementia and AD [111]. Different animal models with AD have displayed encouraging results from voluntary exercise training. It has been found that voluntary wheel running for 16 weeks could result in an improved capacity for exploring novel objects in a recognition memory paradigm when compared with forced exercise and sedentary controls in a Tg2576 mouse model [112]. In a transgenic APOE4 animal model aged 10–12 months, voluntary wheel running for six weeks promotes the more-noticeable recovery of cognitive impairment when compared to sedentary counterparts [113]. In addition, five-month voluntary wheel running has been demonstrated to decrease Aβ plaques in hippocampal tissue and improve learning capacity [114]. Tg2576 mice at the age of 17–19 months used as a mouse model of AD reveal a significant cognitive impairment and neuropathology consistent with AD; Kathryn [115] has found that wheel running intervention for three consecutive weeks effectively improved memory, thereby making the mouse models indistinguishable from wild-type mice on all tasks. A previous study using a TgCRND8 mouse model with AD also demonstrated that five-month voluntary wheel running begun at the age of one month improved cognitive performance when compared to the sedentary control group, which supports the hypothesis that an exercise-induced improvement in cognitive capacity if exercise is begun at the young age, prior to the AD pathogenesis. Furthermore, voluntary wheel running for 10 weeks can significantly delay cognitive decline in APPswe/PS1ΔE9 mouse models when compared with the sedentary controls [116]. Similarly, physical activity has been shown to produce positive effects on brain plasticity and regional gray matter volume [117].

### 4.2. Physical Activity Increases Neurogenesis

Reduced neurogenesis has been reported in different transgenic or knock-in mice with Swedish mutation of the APP or PS1 gene, or in double-transgenic mice with APP and PS1 genes. Exercise is beneficial for multiple pathways and can increase neurogenesis. Clinical exercise trials in normal aging populations have shown increased brain volume [118] following exercise. A previous study reported that voluntary wheel running for 10 weeks presented an enhanced level of hippocampal neurogenesis in APPswe-PS1ΔE9 mice [116]. Interestingly, an age-dependent promoting effect from voluntary wheel running on neurogenesis in hippocampal tissues of 18-month-old APP23 AD mouse model has been confirmed, but no promoting effect on neurogenesis in six-month-old control mice [119], suggesting that voluntary physical activity has the ability to upregulate cell proliferation and neuronal differentiation in AD brain.

### 4.3. Physical Activity Enhances Structural and Synaptic Plasticity in Hippocampus

Synaptic plasticity is the biological process of neurons with specific characteristics of changing their synaptic strength to communicate with others for the purpose of learning and memory capacity. Usually, two forms of synaptic plasticity can be measured in the hippocampus. Long-term potentiation (LTP) [120] is in charge of memory formation, depending on protein synthesis and kinase activation, which can be regarded as the major biological mechanisms for understanding the learning and memory processes. In contrast, long-term depression (LTD) is associated with memory clearance or forgetting. At a cellular level, the impairment of learning and memory in AD is associated with a decrease in LTP and an increase in LTD.

Currently, LTP is recognized as a valuable tool for evaluating therapeutic interventions for disorders of the central nervous system due to its close correlation with learning and memory. Previous findings have shown that Aβ oligomers can inhibit LTP in various hippocampal areas involved in learning and memory processes [114,121,122]. It is well documented that regular exercise can produce a positive effect on cognition and synaptic plasticity. Treadmill exercise can increase expression of LTP as the field excitatory postsynaptic potential (fEPSP) slope increases; it can also spike amplitude in DG both in vivo and in vitro, and enhance synaptic plasticity through lowering the LTP threshold [123,124]. In agreement with previous findings, long-term voluntary wheel running for two to four months has been confirmed to significantly increase the process of neuronal survival in female adult C57BL/6 mice, while concurrently enhancing synaptic plasticity and learning and memory performance, as demonstrated through a Morris water maze (MWM) test [125]. However, LTP could not be produced by a six-month voluntary wheel running treatment in 3xTg-AD animals, and regular exercise only reveals the weak protection from the impairment of LTP induction at the CA1-medial prefrontal cortex synapse [126]. More recently, studies on the effects of exercise on bidirectional plasticity have emerged, and it is reported that forced exercise has an evident effect on LTP in the CA1 region of hippocampus in the rats with sleep deprivation and aging and neurodegenerative diseases, but not in healthy rats [127,128].

### 4.4. Physical Activity Regulates Abnormal miRNAs

Currently, it is still difficult to predict whether the observed abnormal miRNA levels in humans are the cause or consequence of AD progression. Studies of miRNA-expression profiles in AD mouse models may be helpful to address these questions. Previous studies have shown that regular exercise can regulate the expression of miRNAs; however, the underlying mechanisms are still unclear [129]. Our recent findings have demonstrated that miR-34a is significantly increased in AD models when compared with the control; however, eight-week swimming training alleviates the abnormal expression of miR-34a in an AD rat model [39]. Neuroinflammation is a high risk of AD, and Toll-like receptor 4 (TLR4) participates in inflammatory responses. Aerobic exercise can significantly alter the expression of inflammatory cytokines and reduce vascular TLR4 levels in APOE-null mice through upregulating miR-146a and miR-126 and downregulating miR-155 [130]. Similarly, aerobic exercise can downregulate miR-143 level in cardiac tissue [131]. In addition, miRNAs such as miR-22, miR-101a, miR-720, and miR-721 have also been identified in murine brains during the aging process [132]. These findings suggest that miRNAs regulated by aerobic exercise may play an important role in AD. Therefore, aerobic exercise may regulate the expression of the above miRNAs, which should be helpful to prevent the progression of AD. Although studies on exercise to improve AD by regulating miRNAs are still at the stage of infancy, and numerous questions remain unanswered, whether or not miRNAs can be used for the diagnosis of AD depends on an elucidation of the precise characterization, specific distribution, and accurate regulation of miRNAs during the progression of AD. Therefore, further exploration of targets, regulatory networks, and functions is highly desired. Moreover, the scanning and identification of miRNAs during exercise intervention of AD will open a novel avenue for the diagnosis, prevention, and therapy of AD.

## 5. Clinical Studies of Physical Activity in AD

In addition to animal studies, a large prospective study has concluded that regular exercise in AD patients delays the onset of dementia and AD [133]. Human APOE maintains synaptic integrity in the CNS, and its allele APOE4 is associated with an early age of onset and increased risk of AD. Several human studies have shown the interactive effects of exercise and the APOE genotype on cognitive decline. Most studies have confirmed that the protective effects of exercise are more robust in carriers of the ε4 allele [134,135,136,137]. In particular, the impact of low activity is stronger in individuals carrying the APOE4 allele. For example, individuals participating at least twice a week in a leisure-time physical activity have 50% lower odds of dementia when compared with sedentary persons. However, there are inconsistent results about the effects of physical activity in patients with AD, and some studies claim that there is a negative correlation between physical activity and cognitive decline [136,138], while other studies report no relationship [139]. According to previous reports, leisure-time physical activity at midlife twice a week can delay the occurrence of AD for two decades in APOE4 carriers [136], whereas physical activity at the late stage of aging has shorter-term beneficial effects in APOE4 non-carriers [140]. Consistent with the above findings, previous studies on patients with mild cognitive impairment or neurological symptoms suggest that physical activity may still have some benefits in the prodromal or early stage of AD. Moreover, physical activity has a greater protective effect against AD and dementia in women than in men [138].

## 6. mTOR as a New Target for the Prevention and Treatment of AD During Physical Activity?

As reported above, mTOR seems to be an interesting candidate target for the regulation of AD, and the role of physical activity as a neuroprotective agent is well recognized. Some literature has also reported that mTOR is a regulatory target of AD during physical activity.

mTOR signaling is dynamically regulated by upstream components including PI3K/Akt, AMPK, mitogen-activated protein kinase (MAPK), p53, liver kinase B1 (LKB1), erb-b2 receptor tyrosine kinase 2 (ERBB2), insulin receptor substrate 1 (IRS-1), phosphatase and tensin homolog (PTEN), GSK-3, and insulin/insulin-like growth factor 1 (IGF-1). PI3K/Akt, AMPK, GSK-3, insulin/IGF-1, and AMPK play a critical role in regulating the generation of Aβ and the aberrant phosphorylation of tau [27,50,141,142]. PI3K-Akt can activate mTOR-mediated biosynthetic processes, whereas it also can also simultaneously repress autophagic degradation. Previous findings have demonstrated that aberrant activation of neuronal PI3K/Akt/mTOR signaling is an early pathogenesis in the brain of AD individuals and a major candidate for pathophysiological change of Aβ. In addition, the abnormal PI3K/Akt/mTOR signaling pathway has been shown to contribute to the development of AD [27]. Based on the relationship between upstream components of mTOR signaling and autophagy, physical activity should be beneficial to the prevention and alleviation of AD through regulating PI3K/Akt and AMPK signaling.

According to previous reports, the hyperactivation of mTOR can suppress autophagy, which directly contributes to hyperphosphorylation and the aggregation of tau protein [43,44]. Thus, the inhibition of mTOR represents one of the major mechanisms benefitting the pathogenesis of AD in the presence of physical activity. Of course, the effect of exercise on mTOR activity depends on the type and intensity of exercise. Jeong et al. [143] have reported abnormal mTOR phosphorylation and impaired autophagy, such as decreased Beclin1 and LC3B, and increased p62 in the cerebral cortex of NSE/htau23 transgenic mice. Interestingly, 12-week treadmill exercise intervention significantly improves learning and cognitive capacity of NSE/htau23 transgenic mice. Mechanically, abnormal mTOR, impaired autophagy, and the hyperphosphorylation and aggregation (Ser199/202, Ser404, Thr231, PHF-1) of tau protein are improved upon exercise intervention. Meanwhile, Antonella has observed a strong activation of the mTOR signaling pathway, and an increase in two mTOR downstream targets, p70S6K and 4EBP1, in both amnestic mild cognitive impairment (MCI) and AD patients when compared with that of the controls [144]. Interestingly, p70S6K and 4EBP1 are dramatically increased in AD, and are also positively correlated with tau phosphorylation [145,146], thus the activation of p70S6K and 4EBP1 has been identified as a contributor to hyperphosphorylated tau. In contrast, the significant autophagy impairment has also been found. These findings suggest that the alteration of mTOR signaling and autophagy occurs at the early stage of AD. Consistent with previous findings, one study has established a relationship between mTOR signal activation and AD, and a possible correlation of mTOR activation with the degree of cognitive impairment in AD [147]. Besides regulating autophagy and mTOR, 12-week treadmill exercise from the age of 24 months has been reported to markedly suppress Aβ-dependent neuronal cell death and upregulate the expression of NGF, BDNF, and phosphor-CREB in the hippocampal tissue of Tg mice [148]. Furthermore, treadmill exercise may specifically repress GSK-3α/β activity via elevated PI3K and Akt phosphorylation in hippocampal tissue. In a 20-week high-fat diet (HFD) rat model, eight-week treadmill exercise significantly decreased tau hyperphosphorylation and aggregation, while increasing insulin signaling-related protein activity [149]. The above findings suggest that treadmill exercise can provide a therapeutic potential to inhibit tau, Aβ-42, and neuronal-death signal pathways. Therefore, treadmill exercise may be beneficial in prevention or treatment of AD.

AMPK, as a key enzyme for energy metabolism, regulates cellular metabolism to maintain energy homeostasis in response to the reduction of intracellular ATP levels. AMPK is activated when cellular ADP level is increased with the accompanying changes in cellular energy status [150]. AMPK has been implicated in aging and neurodegenerative diseases [151,152]. In addition, AMPK also participates in the regulation of Aβ level and limits the generation of Aβ by inducing autophagy [141,153]. Increasing data have demonstrated the close relationship between AMPK signaling and major hallmarks of AD [154,155,156,157]. T2MD is a risk factor for AD, and diabetic populations at the midlife stage carry a 1.5-times higher risk for developing AD than those diagnosed with T2DM at a late stage in life [158]. Impaired insulin sensing in the brain, diabetes, and metabolic syndrome (MetS) are associated with the pathogenesis of AD, MCI, and other neurological disorders [159]. One recent study [160] demonstrated that mixed intervention, such as nutritional ketosis combined with high-intensity interval training (HIIT) (in order to inhibit mTOR signaling) for 10 weeks, can significantly reduce HgA1c, fasting insulin, and insulin resistance, as well as restore memory function, improve neuroplasticity, and normalize MetS biomarkers of patients via activating the AMPK signaling pathway. This finding suggests that mTOR suppression and AMPK induction may functionally halt neurological disease progression and restore early-stage memory loss. In addition, previous studies have reported mTOR as a target of physical activity in triple-negative breast cancer (TNBC), and physical activity at moderate to vigorous intensity induces the inhibition of PI3K-Akt-mTOR signaling and slows the growth of TNBC cells [161,162,163].

Up to now, the underlying mechanisms of physical activity for mediating these benefits have remained unclear. The neurophysiological effects of physical activity and regular exercise are thought to be mediated by various molecular mechanisms, including the upregulation of BDNF, IGF-1, and related molecules such as Ca^2+^/calmodulin-dependent protein kinase II (CaMKII) and calcineurin, which are associated with learning and memory functions and can, in turn, enhance brain plasticity and improve performance of memory tasks. Forced treadmill running for five days can induce an increase of BDNF protein level within the brain tissues of animals by 70%, which is associated with the increased activation of BDNF receptors and subsequent mTORC1 signaling in hippocampal tissue [144]. Another study has also explored the effect of regular exercise on the upregulation of BDNF, the phosphorylation of BDNF receptors such as tropomyosin-related-kinase (Trk), and the activation of PI3K/Akt [145]. Reelin is an extracellular, secreted glycoprotein that is essential for neuronal migration, synaptic plasticity, and brain development. During the development of the brain, regular exercise increases the production of reelin [146]. In addition, regular exercise can shift the redox state of the brain. Previous studies have also confirmed the minimal change of lipid peroxidation in hippocampal tissue after regular exercise training [37]. Interestingly, BDNF also possesses metabotropic properties besides its neurotrophic effect. BDNF can upregulate expression of AMPK, ubiquitous mitochondrial creatine kinase (uMtCK), and uncoupling protein 2 (UCP2) [164]. Thus, it is reasonable to suggest that low expression or activity of BDNF can significantly lead to the alteration of these metabolic factors, thus eventually disrupting learning and memory functions. Meanwhile, AMPK, as an activator of autophagy, can slow down the progression of AD [153]. According to the data that the Aβ level in an AD brain is determined by the overall functional status of autophagy, AMPK activation can facilitate the triggering of autophagy and promote lysosomal degradation of Aβ through suppressing mTOR signaling.

In this review, we have reported that physical activity not only can attenuate cognitive impairment, but also inhibit the generation of Aβ in different AD models. What is more important, physical activity can induce autophagy in AD rats and mice. Furthermore, physical activity can significantly decrease expression of PI3K, p-Akt, and mTOR at the protein level, respectively. Taken together, AMPK/mTOR signaling may improve insufficient energy metabolism and execute the clearance of Aβ and NFTs via the autophagy signal pathway. Physical activity can inhibit Aβ generation and induce autophagy by downregulating the PI3K/Akt/mTOR signaling pathway, and further can reveal a neuroprotective effect. It seems that physical activity might be a candidate as a neuroprotective agent for AD treatment by inducing autophagy.

## 7. Conclusions and Future Perspectives

**Figure 1:**
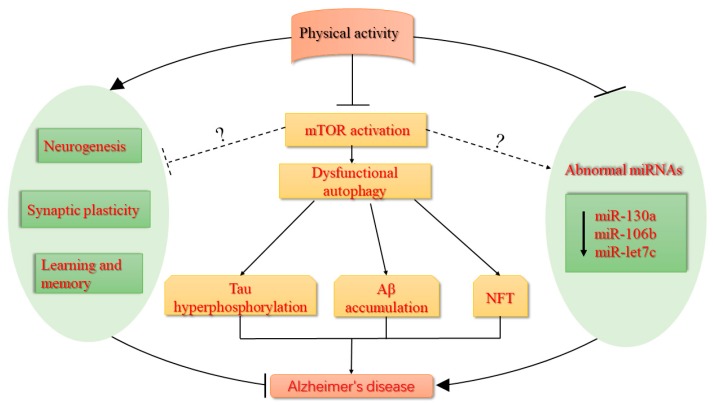
Physical activity as an mTOR suppressor can alleviate cognitive dysfunction and rescue abnormal miRNAs in AD for regulating functional status of autophagy, tau hyperphosphorylation, and the accumulation of Aβ and NFTs, thus accomplishing the mitigation of AD. Meanwhile, mTOR could be considered as the preventive and therapeutic target to develop novel and effective intervention strategies for AD and other neurodegenerative diseases. The solid arrows present the activation and the dotted arrows present the suppression, as well as question symbols presents the uncertainty.

AD is one of the leading aging-related diseases worldwide due to its high rate of mortality and disability. Taking into account the scarcity of effective therapy for AD, developing novel and effective preventive or therapeutic exercise-based strategies based on these novel biological targets is highly desirable. Not all of these studies on regular exercise or physical activity have clearly elucidated a beneficial effect on AD, but regular exercise or physical activity should still be a potent preventive or treatment strategy of AD. Physical activity can alleviate cognitive dysfunction of AD through suppressing mTOR signaling pathways and rescuing abnormal expression of miRNAs, thereby regulating the dysfunctional status of autophagy, tau hyperphosphorylation, and the accumulation of Aβ and NFTs, and ultimately mitigating AD. However, the relationship among regular exercise or physical activity, mTOR suppression, neurogenesis and synaptic plasticity, and rescuing abnormal microRNAs still needs to be further explored in brain tissues, as summarized in Figure 1. Meanwhile, mTOR could be considered as the preventive and therapeutic target to develop novel and effective intervention strategies for AD and other neurodegenerative diseases.

How does regular exercise or physical activity initiate these neuroprotective effects in the CNS? Up to date, this is an intriguing question with no definitive answers. Now, the challenge is to address the cause-consequence relationship between miRNA dys-regulation and AD pathogenesis, and whether the changes in miRNA expression can contribute to AD pathogenesis. Therefore, future works for establishing the link with certainty are highly desired. Meanwhile, the following aspects should be conducted: (1) optimal exercise intervention should be screened according to behavioral results; (2) target miRNAs in serum and hippocampal tissue during exercise intervention of AD should be screened and identified; (3) the manner in which physical activity regulates target miRNAs and autophagy for regulating AD should be further explored and elucidated.
